# How Does Protein Zero Assemble Compact Myelin?

**DOI:** 10.3390/cells9081832

**Published:** 2020-08-04

**Authors:** Arne Raasakka, Petri Kursula

**Affiliations:** 1 Department of Biomedicine, University of Bergen, Jonas Lies vei 91, NO-5009 Bergen, Norway; petri.kursula@uib.no; 2 Faculty of Biochemistry and Molecular Medicine & Biocenter Oulu, University of Oulu, Aapistie 7A, FI-90220 Oulu, Finland

**Keywords:** myelin, myelination, development, peripheral neuropathies, protein folding, transmembrane protein, protein-membrane interaction, protein-protein interaction

## Abstract

Myelin protein zero (P0), a type I transmembrane protein, is the most abundant protein in peripheral nervous system (PNS) myelin—the lipid-rich, periodic structure of membrane pairs that concentrically encloses long axonal segments. Schwann cells, the myelinating glia of the PNS, express P0 throughout their development until the formation of mature myelin. In the intramyelinic compartment, the immunoglobulin-like domain of P0 bridges apposing membranes *via* homophilic adhesion, forming, as revealed by electron microscopy, the electron-dense, double “intraperiod line” that is split by a narrow, electron-lucent space corresponding to the extracellular space between membrane pairs. The C-terminal tail of P0 adheres apposing membranes together in the narrow cytoplasmic compartment of compact myelin, much like myelin basic protein (MBP). In mouse models, the absence of P0, unlike that of MBP or P2, severely disturbs myelination. Therefore, P0 is the executive molecule of PNS myelin maturation. How and when P0 is trafficked and modified to enable myelin compaction, and how mutations that give rise to incurable peripheral neuropathies alter the function of P0, are currently open questions. The potential mechanisms of P0 function in myelination are discussed, providing a foundation for the understanding of mature myelin development and how it derails in peripheral neuropathies.

## 1. Introduction

Myelin is required for axonal saltatory conduction in the central and peripheral nervous systems (CNS and PNS, respectively) of vertebrates [1]. While involved in the structural and trophic support of the axon itself [2,3,4,5], the insulative character of myelin arises from a highly specialized plasma membrane, which is wrapped around selected axonal segments through a mechanism powered by actin disassembly [6]. After several dozen wraps, myelin-specific proteins trigger compaction of myelin, forming a highly periodic lipid-rich structure with very low water content of only ~40% of the total myelin mass [7,8,9]. In the PNS, each separate myelin unit along an axon originates from a single glial cell, the Schwann cell, which matures from a Schwann cell progenitor cell (SCP). These in turn arise from the neural crest during early post-natal development along with other glial cells [10]—a proliferation pathway that is not present in the CNS. Oligodendrocytic myelination in the CNS is arranged differently to that in the PNS [11], although the mature myelin sheaths in both the CNS and PNS morphologically resemble each other [12].

The greatest biochemical difference between the multilamellar (i.e., compact) membranes of CNS and PNS myelin lies in their respective proteomes [13,14]. CNS and PNS myelin are highly enriched in a mere handful of proteins, which is an unusual feature in most biological systems. Many of these proteins are multifunctional [15,16,17,18,19,20,21], contain large stretches of intrinsic disorder [12], tolerate highly hydrophobic conditions [22], and have extended lifetimes [23]. While there is some overlap between the CNS and PNS myelin proteomes [13,14], proteins that are specific either to the CNS or the PNS are present especially in compact myelin, which forms the insulative bulk of the myelin sheath. CNS- and PNS-specific proteins that do not share sequence homology appear to perform overlapping roles in stabilizing compact myelin [24]. The dominant compact myelin proteins in the CNS are myelin basic protein (MBP) and proteolipid protein (PLP) [13,14], whereas in the PNS, myelin protein zero (P0, also known as MPZ) constitutes the largest protein fraction, mostly accompanied by MBP and peripheral myelin proteins 2 and 22 (P2 and PMP22, respectively) [14,25]. All of these proteins are involved in adhering apposing membrane leaflets together [24,26,27,28], forming the fundamental basis of compact myelin stability.

Compact myelin must remain stable to ensure myelin-facilitated insulation; mutations that compromise the structure or function of myelin proteins, or autoimmunogenic events that involve these proteins may result in crippling incurable diseases that involve demyelination—the systematic destruction of myelin and its insulative ultrastructure. These conditions include peripheral neuropathies in the PNS, such as Charcot–Marie–Tooth disease (CMT) and Dejerine–Sottas syndrome (DSS) [29,30], and multiple sclerosis in the CNS [31]. Over 70 CMT and DSS mutations have been described for P0 alone [32]. Some mutations severely truncate P0, some act by disturbing the molecular mechanisms of P0-mediated myelination, and some by triggering the unfolded protein response (UPR) [33,34,35], which likely arises from P0 misfolding or other related mechanisms that cannot be cleared by endoplasmic reticulum (ER)-associated protein degradation (ERAD) [36]. 

The narrow extracellular space between pairs of compact myelin membranes, the intramyelinic compartment, is compacted by a densely arranged P0 zipper in the PNS, which dictates the stability and width of this compartment [28,37,38,39,40,41]. While numerous studies have specifically focused on the compaction of the intramyelinic compartment, P0 has also been shown to stabilize membrane stacking in the narrow cytoplasmic compartment [42,43]; this is supported by recent biophysical experiments using model membranes [41,44]. In animal models, the stacking occurs in the absence of cytosolic MBP and P2 [42,45,46], which poses open, neglected questions, as cytoplasmic membrane leaflet adhesion by P0 remains poorly understood. In addition, the basal expression of P0 before the onset of myelination raises doubts about the apparent redundancy of P2 and MBP [47], especially since MBP is an essential membrane stacker in CNS myelin [17], and both MBP and P2 are required for the long-term stability and the correct ultrastructure formation of PNS myelin [46,48]. These questions will serve as the major focus of this short review, which aims to motivate further studies in the field—especially those that are not dependent on simplified model systems—in order to establish a complete model of P0 and its role as an executive factor of PNS myelination.

## 2. The Molecular Structure of P0

**Figure 1:**
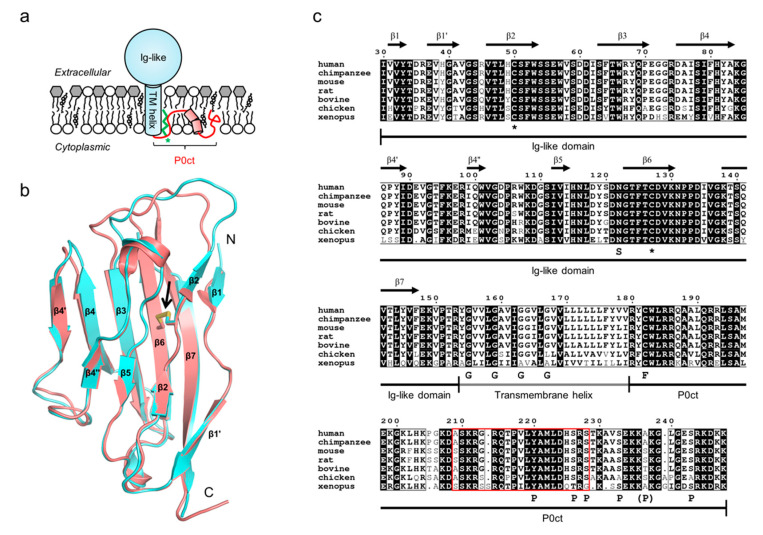
The molecular structure and conservation of P0. (**a**) The domain architecture and membrane topology of P0. The individual protein domains are labeled, with the highly positively charged P0ct highlighted in red. Fatty acylation of P0ct is shown in green, with the position Cys182 in P0ct indicated with an asterisk. Phospholipids and glycolipids are shown with white and gray headgroups, respectively. TM, transmembrane. (**b**) Structures of the rat (blue; PDB ID 1NEU [37]) and human (red; PDB ID 3OAI [40]) P0 Ig-like domains. The conserved disulfide bridge is indicated with an arrow. (**c**) Sequence alignment of the predominant P0 isoform from selected vertebrates. The signal sequence has been omitted, and the numbering and secondary structure labels correspond to human P0. The disulfide-linked cysteines (asterisks), the fatty acylation site (F), the glycosylation site (S), and the Gly zipper (G) motif in the transmembrane domain are indicated. Known phosphorylation sites are labelled (P). The P-site in brackets is phosphorylated in mice but not conserved in humans [66]. The lines below the alignment denote the individual P0 domains. The P0ct neuritogenic segment is represented by the red box.

P0 is a plasma membrane-localized type I transmembrane protein that consists of a 120-residue N-terminal extracellular immunoglobulin (Ig)-like domain, a single 30-residue transmembrane helix, and a 70-residue C-terminal cytoplasmic tail (P0ct) (Figure 1a). P0 exists predominantly as a single translated isoform, although recently a longer isoform (L-MPZ) of unknown function has been described. L-MPZ is produced through a stop-codon readthrough and as such differs in the length of the C-terminal extension [49,50]. Upon expression, P0 and L-MPZ are produced as precursors that contain a 30-residue N-terminal signal peptide sequence, which is cleaved off during membrane insertion [51]. The only known close homolog of P0 is MPZ-like protein 1 (MPZL1, also known as PZR [52]), which is expressed in many tissues outside the PNS and functions as a plasma membrane-bound signaling receptor [52,53,54].

The Ig-like domain is the only part of P0 that has been characterized at high resolution (Figure 1b) [37,40]. The domain is a pair of β-sheets stabilized by a single intramolecular disulfide between two conserved cysteine residues (Figure 1b,c) [37,40,55]. The Ig-like domain is structurally very similar to the Ig-like domain in MPZL1, although they share only ~46% sequence identity [56]. P0ct differs drastically from the Ig-like domain, as it has a compositional bias typical for intrinsically disordered proteins (IDPs) [57] and is highly positively charged, much like MBP [12,17]. Systematic biophysical characterizations using simplified lipid compositions have been performed on P0ct, which revealed that P0ct alone is an IDP in solution but folds upon irreversible association with negatively charged detergent micelles and lipid vesicles [58,59,60,61]. P0ct embeds deep into the lipid structure and affects the mechanical and thermodynamic properties of its surroundings [41,44]. This association in simplified model systems is influenced by ionic strength and the presence of Ca^2+^ [62], and it generally resembles the behavior of MBP under similar conditions, which involves a large gain of structural content [41,58,59,60,61,63]. The association is of electrostatic origin, and the subsequent insertion is thought to arise from the charge neutralization of P0ct by the negatively charged membrane field, which would allow P0ct to fold and potentially display surface chemistry that allows it to interact with the hydrophobic lipid tails. The formation of amphipathic structures is possible, but further structural studies are required in this respect. Another interesting aspect is the ability of MBP to interact with the cytoskeleton, and P0ct has historically been hypothesized to do the same [64]. Generally, P0 is highly conserved throughout vertebrates, especially in mammals (Figure 1c). P0 has been shown to undergo dimerization *via* forming Gly zippers through a conserved motif of evenly spaced Gly residues in the transmembrane domain [65].

P0 is a target for post-translational modifications (PTM). The Ig-like domain contains a conserved N-linked glycosylation site, which commonly displays the human natural killer-1 (HNK-1) epitope [67], a major glycan present in the nervous system. This is a prominent modification of the Ig-like domain [68], given its relatively small size of ~120 residues. In P0ct, a conserved cysteine, Cys182, is a fatty acylation site and most often palmitoylated [69,70]. This modification is essential for the formation of compact myelin and likely to anchor P0ct to the membrane, enhancing its association and increasing the half-life of P0 [71]. Additionally, P0ct contains numerous phosphorylation sites, including the YAML motif (residues 220–223) and several Ser residues in the C-terminal region of P0ct [66,72,73,74,75]. It is noteworthy that P0ct harbors a neuritogenic sequence, which in rodents, when injected as a peptide, induces experimental autoimmune encephalomyelitis [76]. This peptide adopts a kinked, partially oriented helical conformation under membrane-like conditions that are based on simple model lipid compositions [41].

## 3. P0 is the Executive PNS Membrane Stacker

**Figure 2:**
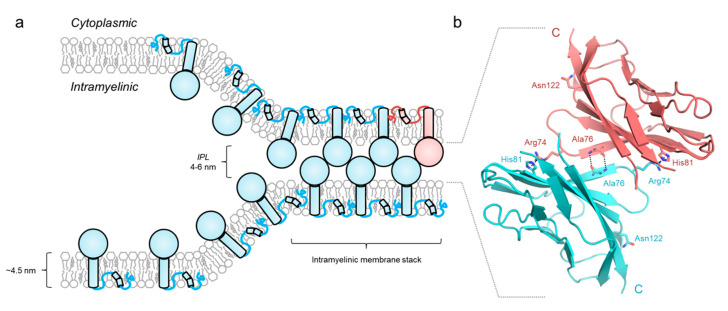
The arrangement of P0 in the IPL. (**a**) Homophilic intercalation of P0 Ig-like domains forms the basis of membrane stack stability in the PNS intramyelinic compartment. (**b**) Structural basis of the homophilic interaction between two apposing Ig-like domains. The intermolecular interaction occurs between the β4 strand backbone atoms of each Ig-like domain and between Arg74 and His81, and is based on the crystal packing of rat P0 (PDB ID 1NEU [37]). The dashed lines (black) represent hydrogen bonds between the backbone atoms of Ala76 and the ion-dipole interactions between Arg78 and His81 sidechains of each Ig-like domain. The conserved Asn122 glycosylation site is shown.

In PNS myelin, P0 is responsible for the formation of the intraperiod line (IPL)—the 5-nm narrow intramyelinic compartment, where apposing Ig-like domains adhere to one another, bringing the two myelin membranes together [42,77,78]. During PNS myelination, the first close intermembrane contacts are formed by the extracellular leaflets of the Schwann cell membrane [79,80,81]. The initial intermembrane distance is somewhat larger than in compact myelin, and upon compaction, the shortening of this distance to form the double intraperiod line held together by P0 may involve e.g., the rearrangement of apposing P0 Ig-like domains [68], the removal of myelin-associated glycoprotein [82], or the degradation of large glycans [83]. Initially, the periodicity of myelin was described in early electron microscopy (EM) and diffraction experiments [7,84,85,86]. Later, this periodicity was found to stem from the presence of P0 [42], and the crystal structure of the P0 Ig-like domain provided atomistic clues into the molecular architecture [37], which fell under scrutiny of modeling approaches that also considered the swelling of the IPL in different ionic conditions and pH values [38,87,88]. In early EM studies, the IPL was resolved as a distinct double structure [89], and recently, a zipper-like framework of isolated bovine P0 reconstituted in model lipid membranes was described using cryo-EM, whereby apposing Ig-like domains from either model membrane are in close contact, with lateral P0 molecules being more separated (Figure 2a) [41]. Similar homophilic interaction was observed in the rat P0 extracellular domain crystal structure [37], and an arrangement with hydrogen bonds between the backbone atoms of Ala76 of two apposing Ig-like domains has been proposed. In this scheme, the main adhesive property arises from an interaction between Arg74 and His81 of the first and second Ig-like domains, respectively (Figure 2b) [38]. In this setting, the C-terminal end of the Ig-like domain, which is followed by the transmembrane helix, would face the membrane [41]. Both Arg74 and His81 are highly conserved (Figure 1c) [28], and the H81R mutation is linked to severe forms of CMT, directly reducing the adhesive capabilities of P0 [90,91,92]. Mutations in Ala76, as well as the adjacent Asp75, are also linked to CMT [32]. The described intermolecular interactions combined with stabilizing lateral interactions could lead to the formation of large adhesive protein surfaces and be sufficient for forming the IPL [41]. The Ig-like domain is glycosylated at Asn122 [67,93], which sits close to the membrane and could interact with glycolipids or adjacent P0 molecules. This might have relevance in the overall positioning of the Ig-like domains for productive adhesion, as suggested earlier through molecular modeling studies [68]. Indeed, the removal of Asn122 and addition of new glycosylation sites in the Ig-like domain have been linked to adhesion loss and CMT [93,94,95], as will be discussed below.

The lateral oligomeric arrangement of P0 remains to be accurately determined, despite several studies having focused on the oligomerization of P0 [37,39,43,96]. The oligomeric state has been suggested to arise from the dimerization of transmembrane domains *via* an internal Gly zipper, which is conserved among terrestrial vertebrates (Figure 1c). Furthermore, the CMT-linked G164R mutation, located in the middle of the Gly zipper, abolishes dimerization [65]. However, this Gly residue is not present in *Xenopus laevis*, yet its P0 retains the ability to form dimers and tetramers that are resistant to various denaturants and detergents, and remains stable over a wide pH range [39]. An ultrastructural architecture involving dimerized Gly zippers has been proposed [65]. This model assumes the presence of lateral tetramers of Ig-like domains; in recent cryo-EM studies, monomeric Ig-like domains were observed with constant lateral spacing in myelin-like stacks composed of model lipid membranes [41]. On the other hand, P0ct might also be involved in lateral oligomerization, as its folding is altered between different detergents, much like the oligomeric state of full-length P0 [41]. Additionally, P0ct was found to affect the homophilic adhesion of the Ig-like domains in transfected non-myelinic cells expressing P0 [97].

P0 is particularly important for PNS myelin, as its absence compromises the formation of compact myelin [98,99,100]. Mouse models lacking P0 cannot be rescued by PLP—the highly abundant transmembrane protein thought to be a major contributor to intramyelinic membrane stacking in the CNS [100]. This is not the case in the opposite setting, and P0 is able to replace PLP to some extent in the CNS [101]. However, replacement of PLP with P0 in the mouse CNS has two remarkable consequences: (1) the CNS IPL spacing increases to a similar width as in the PNS, and (2) Schmidt-Lanterman incisures (SLIs), the PNS-specific transversal veins of cytoplasm within compact myelin, can also form in CNS myelin [102]. Therefore, while P0 is normally not present in SLIs, its presence is required for SLI formation. The roles of PLP and P0 have co-evolved [103], and while several P0 isoforms are present in both CNS and PNS myelin of certain cartilaginous fish [104], it has eventually been lost from the CNS in terrestrial vertebrates and been replaced by PLP among other proteins [103,104]. Normal SLI-like cytoplasmic veins in the CNS are present in significantly lower numbers than SLIs in the PNS [101,102], mostly forming longitudinally [105], but also transversally [106], and their formation is regulated by MBP and 2′,3′-cyclic nucleotide 3′-phosphodiesterase [107]. In addition to P0, MBP levels also control SLI morphology in the PNS [48], revealing an overlapping function. This is of interest due to the similar physicochemical properties of MBP and P0ct.

The Ig-like domain of P0 has been found to interact with PMP22 [108,109], an abundant tetraspan protein in PNS myelin, and the interaction might have relevance in membrane stacking, as a specific ratio of P0 to PMP22 is required to maintain normal myelination [110]. PMP22 resembles PLP topologically, and it belongs to the claudin family of adhesion proteins [111,112]. PMP22 has been demonstrated to act as a membrane stacker [113], but PMP22 cannot rescue myelination when P0 is missing [114]. The periodicity of PNS myelin varies with pH [115], and comprehensive experiments establishing the role of electrostatics and proteins in periodic variation that stem from pH and ionic strength have been conducted [87,88], suggesting that the swelling of the IPL in PNS myelin largely depends on the abundance of negatively charged residues in the P0 Ig-like domain. This, however, was concluded before the structural characterization of the domain, and now, the width of the IPL is thought to be mostly governed by the apposition of Ig-like domains, as illustrated in Figure 2a. Recently, the effect of acidic pH was suggested to be a consequence of P0 Ig-like domain denaturation [116], and the observed closer packing of the membranes that form the IPL at low pH could reflect PMP22 being able to maintain a compact IPL. This suggests that IPL stability after myelin formation might not immediately require P0, but future studies are certainly required to shed light on the matter. PMP22 has been linked to the formation of lipid rafts in Schwann cells [117], and P0 is known to localize to rafts of certain lipid composition [118]. Moreover, P0 trafficking is dependent on membrane cholesterol content [119] (see below).

Due to available structural information, the Ig-like domain and its role in defining the spacing of the IPL have been the focus of several studies. In contrast to the literature published on the Ig-like domain and IPL formation, studies focusing specifically on the structure-function relationships of P0ct account for a small fraction of experimental data available for P0 [41,44,58,59,60,61]. MBP and P0ct share similar physicochemical characteristics, which potentially confers P0ct with MBP-like functions. These might include a role in the formation of cytoplasmic cavities [107]. Importantly, based on in vitro studies on how P0ct interacts with model lipid bilayers, P0ct might mediate the packing between membranes that form the cytoplasmic compartment [41,44]. Central results supporting this hypothesis arose from experiments where P0ct irreversibly associated with simplified model membrane systems with a similar affinity as MBP [41,44,63] and aggregated lipid vesicles together, producing membrane spacings typical for the MDL [41,44]. The effect was dependent on the dosage of P0ct, the net charge of the lipids, ionic strength, and the presence of Ca^2+^ [41,44,62]. Similar chemistry, however, might not be sufficient alone: for the ability to stack phospholipid membranes, a structural architecture that enables stacking must be achieved. In the case of MBP, this architecture has been subject to decades of research [17]. While evidence obtained through comparison of PNS myelin isolates from wild-type and MBP-deficient *shiverer* mice suggested that MBP would only bind to the membrane headgroups [120,121], a range of subsequent biophysical studies indicate that MBP also partially inserts into the bilayer core [122,123,124,125,126,127]. The accurately determined protein-free structure of the myelin lipid bilayer [128] is altered in this process [63,124]. Based on earlier literature and recent experimental data, a membrane stacking mechanism for MBP has been proposed [63], although further confirmatory studies using other experimental approaches are certainly required, especially those that involve isolated natural myelin.

**Figure 3:**
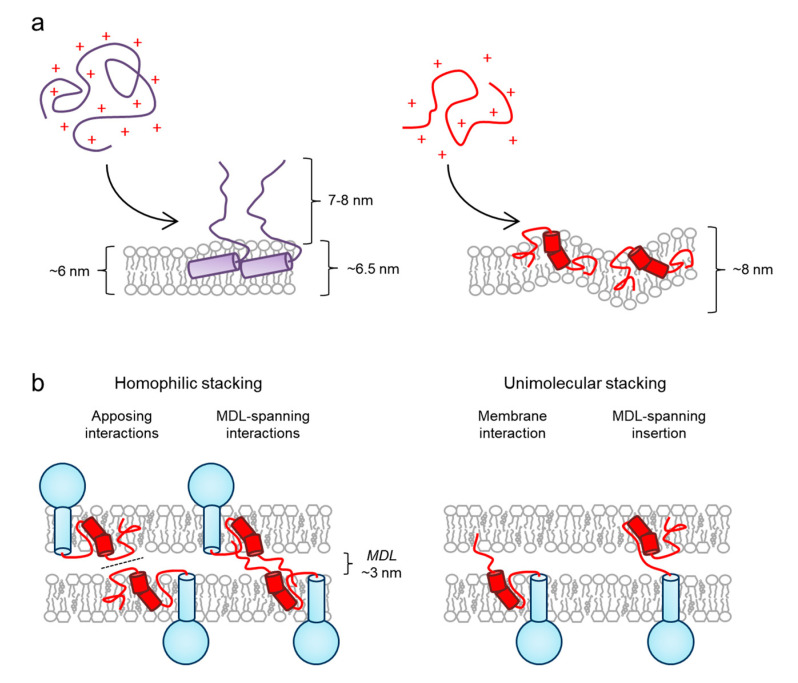
Hypothetical membrane stacking architecture of P0ct in the MDL. (**a**) The association of the positively charged MBP (purple; left) and P0ct (red; right) with a single phospholipid bilayer is different, as shown using model systems employing simple lipid mixtures: P0ct fully embeds into a membrane, and by doing so promotes membrane undulation and the liquid-disordered phase [44,63]. (**b**) Possible arrangements of P0ct and two apposing bilayers in a membrane stack. P0ct is drawn in red with the rest of P0 in blue.

Experimental evidence suggests that P0ct differs from MBP in one noteworthy aspect during membrane association: when MBP binds to a single membrane, a partially folded intermediate state is formed, which can further adhere to a second membrane given that MBP reaches a critical concentration [12,63]. On the other hand, P0ct as a free polypeptide completely embeds into model membranes (Figure 3a) [44]; however, more studies regarding the binding mode are needed. This association is supported by the fact that P0ct is kept very close to the membrane in vivo, not only by the transmembrane domain, but also through the fatty acylation of Cys182 [61,71]. Since P0ct is roughly half of the size of MBP, the ability of P0ct to span the MDL is lower compared to MBP, suggesting that P0ct may stack membranes through a different molecular arrangement than MBP. This is also supported by past literature describing a slightly widened MDL in the PNS of *shiverer* mice, which lack functional MBP [77].

Different membrane stacking modes might exist for P0ct in the MDL (Figure 3b). The stacking mode may be homophilic, which is possible given the amount of P0 in PNS myelin. P0ct being enriched in otherwise negatively charged membranes would neutralize the membrane charge, which could result in weak attractive interactions between adjacent P0ct segments. Another mechanism could include P0 molecules swapping their tails into the apposing membrane, allowing P0ct to span the MDL and thus define its width. In the absence of experimental data, unimolecular stacking involving a single P0 molecule that interacts with an apposing membrane cannot be excluded. Regardless of the exact mechanism, P0ct can certainly bridge lipid bilayers together at least in vitro [41]. Importantly, a missense mutation linked to a CMT phenotype with abnormally dense and thick myelin sheaths, D224Y, showed increased activity in biophysical experiments, in which mutated P0ct interacted with vesicles composed of synthetic lipids [44].

When considering the formation of the MDL, MBP is an indispensable component of CNS myelin [17], being present in moderate amounts in the PNS [14]. P2, the small β-barrel that may function as a lipid transporter in myelin maintenance [46], is similarly capable of stacking membranes at a near-crystalline level of order in vitro [21,129]. Unlike MBP and P0ct, however, P2 binds membrane surfaces in a reversible manner [21,130], which could enable P2 to have a more transient role in compact myelin. This has implications e.g., during the maintenance of myelin via lipid replenishment, which is a pre-requisite for the long-term integrity of myelin [131,132,133,134,135]. P2 is an important component of PNS myelin, as several mutations lead to CMT [136,137,138,139,140] by affecting the structure-function relationships of P2 [141]. MBP and P2 stack membranes synergistically in vitro [46]. Despite these observations, mice deficient in both MBP and P2 develop normal-appearing PNS myelin [46,48], whereas the lack of P0 results in a severe dysmyelinating phenotype with large non-compacted ultrastructural regions [98,99,100]. This does not imply that MBP is redundant in PNS myelination; mice lacking both P0 and MBP completely lack the MDL, while the presence of MBP in P0-deficient animals produces a slightly less dramatic phenotype [142]. Furthermore, mutations in P0ct that directly interfere with its structure-function relationships or PTMs influence its function in myelin, and thus the myelin phenotype [44,72,143,144]. It is therefore plausible that P0ct functions as the executive membrane stacking molecule in the cytoplasmic apposition, through its ability to compensate for the functions of MBP and P2 in PNS myelin—at least in the developing nervous system [46]. As soluble proteins, MBP and P2 are completely translated in the cytoplasm. When MBP performs its membrane stacking function, local translation of free MBP allows spontaneous interaction with membranes, and the formation of compact myelin [63]. On the other hand, when MBP remains soluble, it may have other functions [12,17]. The solubility of MBP distinguishes it from the expression pattern and trafficking of P0; P0ct is likely to always remain membrane-embedded and have less spatial freedom. 

## 4. The Expression and Trafficking of P0

During PNS development, SCPs proliferate and migrate along axons from the neural crest, differentiating into Schwann cells. This process is regulated by a multitude of molecular factors [145], most importantly neuregulin 1 (NRG1). While Schwann cells develop in the presence of other glia and neurons, they are destined to become either myelinating or non-myelinating Schwann cells, mostly through dose-dependent axo-glial NRG1 signaling [146]. The presence of NRG1 type III is of particular importance [147,148], as low NRG1 type III will direct Schwann cells to form amyelin, or Remak bundles, which lack compacted membranes. Conversely, high NRG1 type III levels promote the formation of myelinating Schwann cells, and the degree of myelination directly correlates with the amount of active NRG1 type III [146].

**Figure 4:**
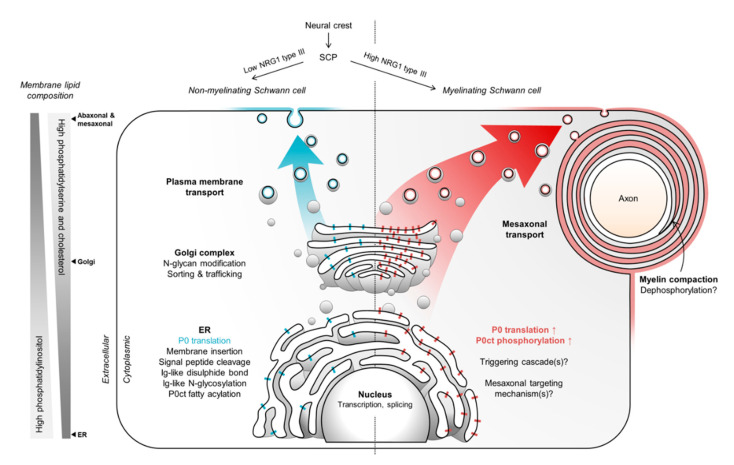
The expression and trafficking of P0 in Schwann cells. SCPs emerge from the neural crest and form non-myelinating Schwann cells under low NRG1 conditions. These cells express P0 at a basal level and target it to the plasma membrane. This process normally does not form myelin-like structures, but in myelinating Schwann cells that proliferate from SCPs at high NRG1 levels, P0 targeting shifts to the mesaxon via unknown mechanisms. Potential myelination-involved factors include intermolecular interactions between P0 molecules, the phosphorylation of P0ct, and/or altered membrane lipid compositions. The changing lipid environment between different membranes affects the partitioning and trafficking of P0 [118,119,150].

The expression and targeting of P0 are illustrated in Figure 4. The translation of P0 begins in the cytosol, and the N-terminal signal sequence targets it to the ER. The signal sequence is cleaved, and most of the P0 chain is translated as it is transported through the membrane. The Ig-like domain folds in the ER lumen, potentially through assisted disulfide bond formation and chaperone activity [55,149]. The transmembrane domain anchors P0 to the membrane, and the final translated segment is P0ct. The fatty acylation of P0ct at Cys182, which increases the lifetime of P0 [71], is likely to occur soon after P0 is fully translated. At this point, P0ct most likely has already entered the membrane, as suggested by recent data [41], especially since the ER membrane is negatively charged due to the presence of phosphatidylinositol [150]. In the ER, P0 is glycosylated at Asn122 [67], followed by its trafficking to the Golgi membranes. Here, the N-glycan of P0 is modified to become mature HNK-1 [67], and P0 is targeted to the plasma membrane [151,152,153]. This trafficking is dependent on at least the phosphorylation of P0ct at Ser228 and Ser233 [73] as well as the presence of a YAML motif [154]. This motif contains Tyr220, which becomes phosphorylated at the peak of myelination [75]. Increased phosphorylation of P0ct, in general, is connected to active myelination [155].

A basal level of P0 can be detected already in neural crest cells and SCPs [156], and in mature Schwann cells, P0 is very abundant [47]. During myelination, P0 is trafficked to the leading mesaxon where it eventually performs its structural function and drives myelin compaction [152]. However, it is important to realize that the presence of P0 in Schwann cells does not imply that myelin has formed in the first place, or will ever form [47]. Ig-like domains will not assemble into zippers and P0ct will not stack membranes only because P0 is present. In fact, P0 functions not involved in myelination have been proposed, including axo-glial crosstalk, as the lack of P0 results in axonal degeneration [98]. Moreover, such functions appear to have specifically evolved for PNS myelin, as replacing PLP with P0 in the CNS produces similar axonal degeneration, which essentially arises from an abnormal phenotype at the myelin paranodes [101,102].

**Figure 5:**
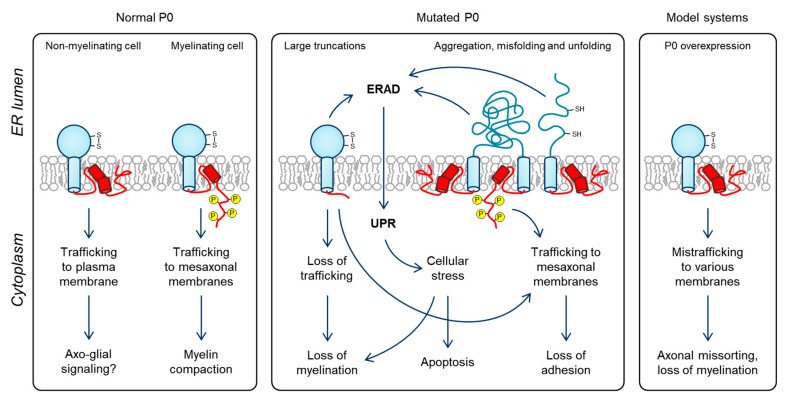
Possible mechanisms of P0 trafficking. Left: In healthy Schwann cells, P0 is either trafficked to the plasma membrane or the mesaxon, depending on whether the cell performs myelination. As P0ct phosphorylation has been linked to myelination, phospho-P0ct might become exposed and function as a mesaxonal targeting signal, and be incapable of membrane stacking before it reaches the nascent myelin multilayer. Middle: P0 mutations result in demyelination through different mechanisms, including loss of trafficking, UPR activation *via* overwhelming ERAD, and loss of adhesion by disturbing the structure and function of P0. Right: in model systems, overexpression of P0 results in P0 targeting to the abaxonal, periaxonal, and mesaxonal membranes, which results in complications that halt myelination. It also affects the sorting of axonal bundles, implying that P0 is involved in axo-glial signaling.

As with MBP in the CNS, the effect of P0 on myelination is dose-dependent [110,157,158], and overexpression of P0 has dire consequences for the sorting of naked axons and the correct formation of myelin (Figure 5) [110,157]. In the mature myelin sheath, P0 is very abundant, and most of it is present in compact myelin. The expression of MBP in the CNS is targeted by transporting the MBP mRNA to where translation is required [159,160,161,162,163]. In the case of P0, other temporal mechanisms that target mature P0 to the mesaxon are required: Golgi-derived vesicles that carry P0 are transported to the spirally wrapped mesaxonal membrane *via* microtubules [151,153]. Thus, elevated expression of P0 needs to be initiated, but at the same time, its trafficking needs to be controlled to avoid crowding in the ER, Golgi, and plasma membranes. Currently, what exactly triggers this expression and trafficking, let alone which factors are involved in the vesicular transport of P0 specifically to the mesaxon, remains unknown. Control of P0 expression is a crucial requirement, as P0 overexpression would result in the halting of myelin wrapping and compaction, and eventually dysmyelination [110,157]. The YAML motif and phosphorylation state of P0ct might be involved, as might the interaction and co-localization of P0 with PMP22, but further studies are required in these aspects. Additionally, how SLI formation and PNS myelination tie together from the perspective of P0 expression and trafficking is another enticing area of scientific interest.

There are essentially two categories of P0 mutations that can induce a demyelinating phenotype: (1) nonsense and frameshift mutations deleting large segments of P0, resulting in loss of structure and function, and (2) missense mutations that disturb the structure and function of P0 required in stable membrane stacks, cause intracellular folding issues with P0, or mislocalize P0. While the effect of nonsense and frameshift mutations is rather trivial, missense mutations can have various effects on P0 [32], which may be immediate or depend on gene dosage [144]. Missense mutations include amino acid changes that result in lost or altered P0 function, such as the H81R mutation discussed above. Another CMT mutation, D224Y, enhances the membrane stacking ability of P0ct in model membrane systems, and it is linked to an unusually dense and thick compact myelin phenotype in affected patients [44,143,144]. Loss of the conserved glycosylation site by the N122S mutation results in a relatively mild demyelinating phenotype [94,164], most likely due to weakened adhesion at the IPL. The S63C mutation has been reported to directly affect the stacking function of P0, resulting in an increased IPL width that stems from dimerizing Ig-like domains via Cys63-mediated intermolecular disulfides [33,165]. Significantly, the effect of this mutation could be reversed through the use of reducing agents, essentially reverting to the phenotype of native-like myelin in mutant animals [165]. On the other hand, missense mutations might also directly influence the trafficking of P0. This could be the case for A221T within the YAML motif, as the mutant P0ct in vitro behaves as the wild-type protein [44], but in vivo, the mutation causes DSS together with a second mutation in the Ig-like domain [166]. In mutations that affect P0 trafficking, the underlying mechanism could arise from either loss of targeting or mistargeting, as in P0 overexpression models [110,157]. Another noteworthy mutation is D61N, which introduces a new glycosylation site in the Ig-like domain, causing myelin outfoldings and partially retained P0 in the Golgi membranes [95]. This suggests that P0 trafficking is influenced either by Asp61 or nearby amino acids, or an increase in glycosylation. The P0 retainment in the Golgi might arise from a slowdown of P0 processing due to the increased level of glycosylation. In turn, the outfoldings might be a direct result of P0 mistrafficking to the abaxonal membrane instead of the mesaxon, resulting in stacking artifacts on the Schwann cell surface.

Several neuropathy mutations in the Ig-like domain and P0ct have been directly linked to UPR [33,34,167,168], which is a condition of the ER that severely stresses the cell and eventually promotes pro-apoptotic mechanisms (Figure 5) [169]. UPR arises from prolonged accumulation of misfolded and unfolded proteins that cannot be cleared rapidly enough through ERAD [170]. ERAD was recently demonstrated to harbor a neuroprotective role in Schwann cell pathology, specifically P0-borne CMT type 1B, as the P0 S63del mutant, which is incapable of folding and accumulates in the ER, is cleared through ERAD [36]. The exact pathological effects of other P0 mutations that induce UPR are subjects for future studies, although the overloading of ERAD may be involved in most cases. Possible molecular mechanisms include misfolding, unfolding, and unspecific aggregation of P0. This includes mutations that affect disulfide bond formation in the Ig-like domain, although a mutation of the conserved disulfide-bonded Cys50 did not impair the membrane trafficking of P0, but caused a lack of adhesion [55]. Another mechanism involves significantly altered P0 expression and/or trafficking patterns that essentially causes nascent P0 to be retained in the ER, overloading its folding machinery. Again, the YAML motif is potentially involved, as the efficient clearance of P0 from the ER would likely be affected if P0 trafficking is blocked or hindered, especially during accelerated P0 expression.

## 5. Future Directions

A key open aspect from an ultrastructural perspective is the oligomerization mechanism of P0. Whether and how P0 forms tetramers, as suggested by earlier reports [37,39,43,65], is a major open question to answer, as Ig-like domain-mediated tetramerization might not be relevant *per se* in the light of recent observations [41]. The transmembrane domain Gly zipper is likely to play a role in oligomerization [65], but the sensitivity of P0 oligomerization to different detergents [39,43] suggests that P0ct could be involved, as its folding in vitro does depend on the choice of detergent [41]. Given the substantial amount of P0 compared to other PNS myelin proteins [14], and the stacking architecture P0 adopts in myelin-like membranes [41], lateral oligomers must exist, but how do they actually form? To assess this, the ambitious goal of acquiring an atomic resolution structure of full-length P0 in a membrane of native myelin composition would help answer the question. Thus far, no reports describing the purification of recombinant full-length P0 exist, while highly pure recombinant P0 will be needed for such studies, instead of protein extracts from native sources. As crystallization might be problematic, a cryo-EM approach will be useful in P0 characterization [41]. The preparation of 2D and 3D ordered samples from highly purified materials should enable the use of hybrid structural biology techniques, including cryo-EM, tomography, atomic force microscopy, or grazing-incidence scattering methods, for unraveling the high-resolution details of P0 assembly in myelin. In an optimal setting, these methods could be employed using lipids derived from natural myelin, which differs substantially in lipid composition from simpler model membrane systems that were employed in the characterization of P0ct and full-length bovine P0 [41]. 

How is P0 trafficked to the mesaxon during myelination? The involvement of the Golgi complex and Golgi-derived vesicles has been established [151,153,157], but the trigger mechanism remains elusive. While the Ig-like domain resides in the lumen of the endomembrane system before reaching the plasma membrane, reports that favor the involvement of the extracellular domain in trafficking are scarce [95]. On the contrary, P0ct is more likely to be involved: not only does it contain the YAML motif involved in trafficking [154]; its multiple phosphorylation states during myelination definitely have a role in bringing P0 to its target compartment [73,75]. In addition, the role of other proteins in the trafficking and function of P0 is still unclear. PMP22 is a known interaction partner and needs to be present in healthy PNS myelin [108,109,110], but other proteins might be involved in the correct targeting of P0, and even harbor synergistic effects when P0 establishes membrane stacks.

P0ct is special in two interconnected aspects. Firstly, it is small and has unique chemical attributes, most notably its high abundance of Arg and Lys that grant it a high positive net charge. Charge-altering modifications, such as phosphorylation, will influence the net charge of P0ct, which in humans contains only 69 residues. Since trafficking signals are commonly found in cytoplasmic domains of membrane proteins, this might regulate P0 trafficking. The known phosphorylation sites in P0ct are clustered within a very narrow sequence stretch [66,72,73,74,75]. Modifying these sites at the correct time during myelination would change the electrostatic properties of P0ct, allowing spatiotemporal regulation of membrane association and potential stacking. Secondly, the lipidic microenvironment seems to determine P0ct behavior in vitro. P0ct associates with model membranes through electrostatic attractions, which arise from both the amino acid sequence of P0ct and the membrane lipid composition. While the ER, Golgi, and plasma membrane cytoplasmic leaflets are rich in negatively charged lipids, the ratio of net neutral and negatively charged lipids varies between each compartment. Additionally, cholesterol and sphingomyelin contents gradually change, while traversing from one subcellular volume to another [150], which directly impacts the formation of membrane lipid rafts [171,172,173]—another aspect influencing P0 localization [118]. Cholesterol is of low abundance in the ER [150], but is a factor of pivotal importance during myelination [174]. Recently, several myelin-specific transmembrane proteins, including PMP22, have been shown to partition into cholesterol-rich membrane domains, and the partitioning appears to be dependent on protein stability [175,176]. Logically, the most important proteins would assemble together with cholesterol, as P0 does [119].

Considering the points made above, it is enticing to speculate how PTMs in P0ct and the gradually changing membrane composition along the P0 trafficking pathway could enable microscopic changes in the affinity and folding of P0ct, as well as its stacking tendency. As biophysical studies focused on the structure-function relationships of P0ct in model lipid environments have recently provided useful information, motivation emerges to study P0ct and P0 in myelin-like lipid compositions, even of natural origin. Therefore, P0ct is a key target for future studies when considering the spatiotemporal targeting of P0 to the mesaxon during myelination. After P0 has reached the mesaxon, a lipid-rich environment, possible phosphatase-enabled activation of densely packed P0 could result in IPL formation and efficient P0ct-mediated MDL stacking, much like how MBP orchestrates MDL assembly in the CNS [63].

## 6. Conclusions

Demyelinating conditions have several etiological pathways with different biomolecular origins. P0 is a fundamental part of PNS myelin and its biogenesis, but also an unfortunate target for CMT and DSS mutations. Many aspects of P0-linked myelination have unveiled in recent years, yet many open questions remain. The lateral P0 assembly, the involvement of P0ct, and the P0ct structure during P0 trafficking and oligomerization are subjects for in vitro and in vivo characterization. Unraveling how P0, together with other compact myelin molecules, achieves its adhesive function at the correct time in the right place is an absolute requirement for understanding the formation and demise of myelin. 

## Abbreviations

CMT	Charcot-Marie-Tooth disease
CNS	central nervous system
DSS	Dejerine-Sottas syndrome
EM	electron microscopy
ER	endoplasmic reticulum
ERAD	ER-associated protein degradation
HNK-1	human natural killer-1
Ig	immunoglobulin
IPL	intraperiod line
MBP	myelin basic protein
MDL	major dense line
NRG1	neuregulin 1
P0	myelin protein zero
P0ct	the cytoplasmic extension of P0
P2	peripheral myelin protein 2
PLP	proteolipid protein
PMP22	peripheral myelin protein 22
PNS	peripheral nervous system
PTM	post-translational modification
SCP	Schwann cell progenitor
SLI	Schmidt-Lanterman incisure
UPR	unfolded protein response
